# Approaches to nanostructure control and functionalizations of polymer@silica hybrid nanograss generated by biomimetic silica mineralization on a self-assembled polyamine layer

**DOI:** 10.3762/bjnano.2.84

**Published:** 2011-11-23

**Authors:** Jian-Jun Yuan, Ren-Hua Jin

**Affiliations:** 1 Synthetic Chemistry Lab., Kawamura Institute of Chemical Research, 631 Sakado, Sakura, 285-0078 Japan; 2 CREST-JST, 631 Sakado, Sakura, Chiba 285-0078, Japan

**Keywords:** biomimetic silica mineralization, linear polyethyleneimine, nanofiber, nanograss, thin film

## Abstract

We report the rational control of the nanostructure and surface morphology of a polyamine@silica nanoribbon-based hybrid nanograss film, which was generated by performing a biomimetic silica mineralization reaction on a nanostructured linear polyethyleneimine (LPEI) layer preorganized on the inner wall of a glass tube. We found that the film thickness, size and density of the nanoribbons and the aggregation/orientation of the nanoribbons in the film were facile to tune by simple adjustment of the biomimetic silicification conditions and LPEI self-assembly on the substrate. Our LPEI-mediated nanograss process allows the facile and programmable generation of a wide range of nanostructures and surface morphologies without the need for complex molecular design or tedious techniques. This ribbon-based nanograss has characteristics of a LPEI@silica hybrid structure, suggesting that LPEI, as a polymeric secondary amine, is available for subsequent chemical reaction. This feature was exploited to functionalize the nanograss film with three representative species, namely porphyrin, Au nanoparticles and titania. Of particular note, the novel silica@titania composite nanograss surface demonstrated the ability to convert its wetting behavior between the extreme states (superhydrophobic–superhydrophilic) by surface hydrophobic treatment and UV irradiation. The anatase titania component in the nanograss film acts as a highly efficient photocatalyst for the decomposition of the low-surface-energy organic components attached to the nanosurface. The ease with which the nanostructure can be controlled and facilely functionalized makes our nanograss potentially important for device-based application in microfluidic, microreactor and biomedical fields.

## Introduction

Silica-based, one-dimensional, nanostructured thin films on substrates with tunable nanostructure and surface morphology are of great importance for various applications, such as photoelectronics [1], high-efficiency sensing and bioanalysis [2–3], protein adsorption [4], cell growth [5], surface wettability control [6] and liquid transformation [7]. Silica nanowire films can be fabricated by catalyst-promoted vapor–liquid–solid (VLS) or solid–liquid–solid (SLS) processes under high temperature conditions, where metallic nanoparticles such as gold, gallium, and tin as catalysts are generally used to improve nanowire nucleation and growth [8–11]. On the other hand, silica films consisted of a network of interwoven nanofibers were also reported as produced by flame spray pyrolysis of organometallic solutions with the deposition and annealing temperature of silica in the range from 500 to 800 °C [12]. However, these conventional methods normally require harsh synthesis conditions, complex techniques or tedious procedures, and also have the disadvantage that it is difficult to produce the nanostructured film on plastics or other low-melting-point substrates due to the high processing temperatures [8–12].

Recently, there has been an increase in the interest in the biomimetic synthesis of silica-based materials, which are characterized by the processing of nanostructured silica in water under mild and ambient conditions, by using either synthetic or biologically-derived amine-containing (macro)molecules as additives, mediators or templates [13–14]. This is completely different from the conventional silica deposition, which requires nonideal conditions, such as elevated temperature, extreme pH, and the presence of either a large amount of surfactants and/or organic cosolvents [15–18]. Moreover, it is essential for practical device fabrication that biomimetic nanostructured silica materials can be directly generated on the surface of various substrates [19]. One method is to chemically bond, or physically adsorb, long-chain amines or proteins on the substrates, in order to form the conformal molecular layer, which then serves as the catalyst for the silica-mineralization reaction on the surface. This method has led to the formation of uniform or particle-based silica thin films on substrate surfaces [20–23]. Based on lithography or printing techniques, silica micropatterning has also been achieved by first patterning silicification-active polymer solutions onto the substrates and subsequently performing silica deposition [24–25]. Cha and coworkers [26] reported using block copolymer thin film for the biomimetic formation of two-dimensional silica nanopatterned arrays. However, the controlled synthesis of silica or hybrid thin films with tunable one-dimensional nanostructures still remains a challenge [20–26].

We are interested in using crystalline, self-assembling linear polyethyleneimine (LPEI) [27] as a mediator for silica deposition under ambient conditions. As one of the simplest synthetic polymers, LPEI is cost-effective and suitable for a wide range of applications. The silica mineralization reaction was assumed to occur site-selectively on the surface of crystalline LPEI aggregates, producing the LPEI@silica hybrid nanostructure or pure silica materials after calcination [28]. Importantly, the attractive feature of this simple approach is that the nanosilicas can be created in a reliable and programmable way with a hierarchical nanostructure and complex morphology [29–31]. Very recently, we found that this crystalline LPEI is further capable of self-assembling into a fibrous layer on the surface of various substrates, which acts as a direct template for the bioinspired formation of LPEI@silica nanoribbons (which we called a “nanograss” surface) after an aqueous and room-temperature mineralization reaction [32–33]. By performing a rapid LPEI crystalline self-assembly on substrates, we also synthesized an ultrathin silica-nanowire-based surface, which demonstrated the feasible modulation of the hierarchical nanostructure and surface morphology [34]. In comparison, the modulation of the nanostructure and surface morphology of nanoribbon-based nanograss films has not been demonstrated in detail [32–33], which is essential for a range of technological applications [1–7].

Herein, we report our approaches for the rational control of the surface nanostructure of LPEI@silica nanograss by adjustment of either the LPEI self-assembly or silica-mineralization reaction conditions. We further demonstrate the use of amine chemistry of free LPEI or LPEI occluded in LPEI@silica nanoribbons for the functionalization of the nanograss film, by selecting three representative examples, chromophore (porphyrin), metal nanoparticle (Au) and oxide (anatase titania). The silica@titania composite nanosurface exhibited an extreme change in photoresponsive wettability due to the presence of photocatalytic anatase titania, which can decompose hydrophobic organic components bonded to the surface.

## Results and Discussion

**Figure 1:**
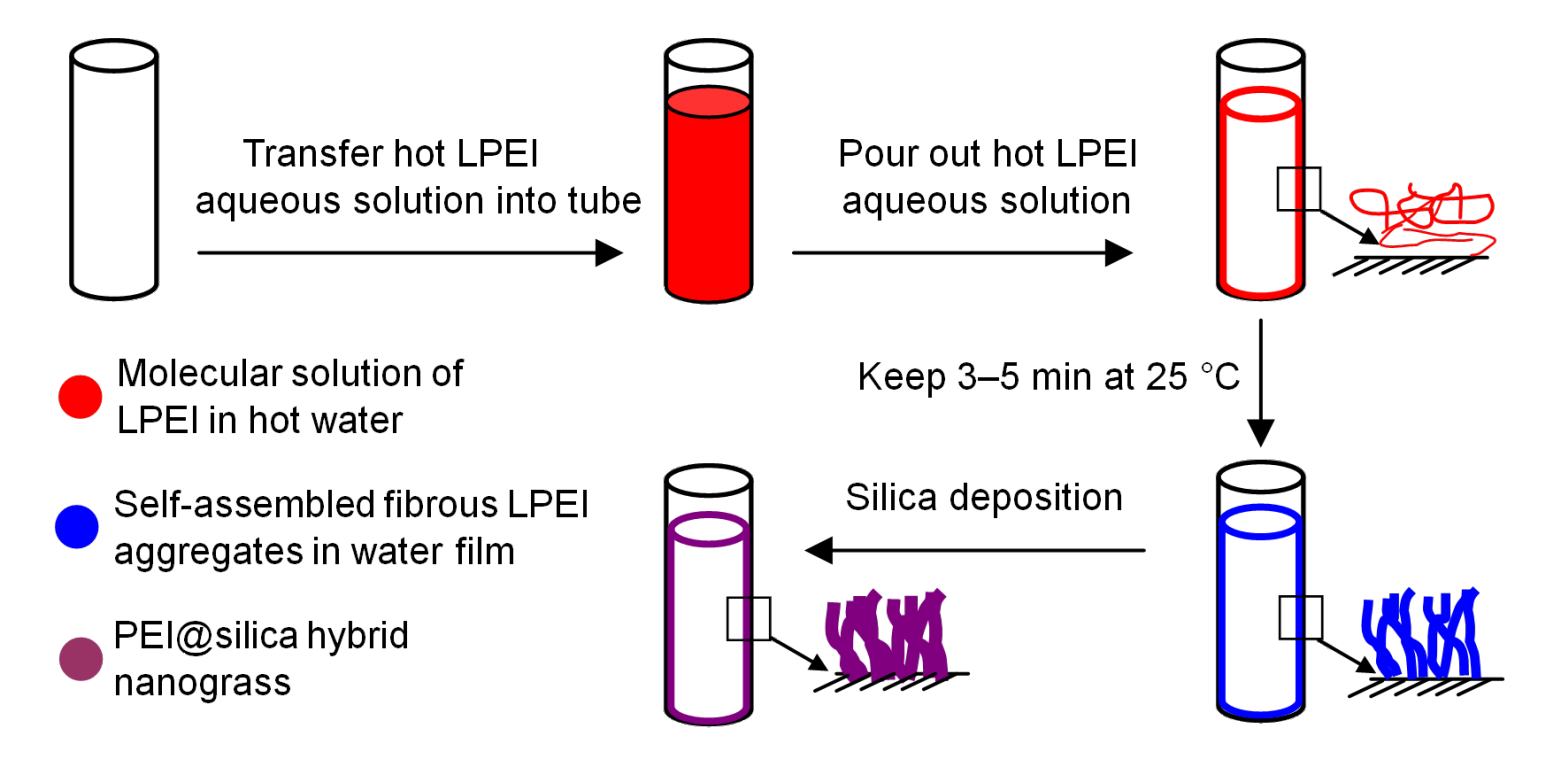
Schematic demonstration of the bio-inspired generation of LPEI@silica hybrid nanograss surface on the inner wall of a glass tube by mineralization of silica on the nanostructured matrix from self-assembled LPEI aggregates.

The inner wall of a soda-lime glass tube was used as a representative substrate for the nanograss generation. Figure 1 demonstrates our process for the generation of a LPEI@silica hybrid nanograss film on a substrate surface. Our process involves i) charging the LPEI hot solution into the tube; ii) pushing out the excess hot LPEI solution from the tube in order to obtain the tube coated with an aqueous layer of molecular LPEI (mLPEI@tube); iii) keeping this tube at room temperature for 3–5 min for crystalline self-assembly of LPEI (cLPEI@tube); and finally iv) immersing the cLPEI@tube into the silica source for biomimetic silica mineralization. The silicification was performed at room temperature for typically 40 min. An aqueous nanostructured LPEI layer on the inner wall of the tube serves as a stable and catalyst-active matrix for the templated-deposition of silica under ambient conditions, leading to the formation of LPEI@silica hybrid nanograss (Figure 1). This mechanism was confirmed by our previous XRD studies, indicating that the crystals from the self-assembled LPEI templates still remained after the silicification reaction [33]. In this work, we demonstrated that the surface nanostructure and morphology of LPEI@silica nanograss can be rationally controlled by adjustment of the synthesis conditions, as summarized in Table 1.

**Table 1:** Summary of surface morphology and nanostructure of LPEI@silica nanograss by adjustment of the conditions for the silica-mineralization reaction and LPEI self-assembly on the substrate surface.

Synthesis conditions		Morphology and structure of nanograss

Media for silica deposition	H_2_O	Dense and well-arrayed nanoribbon structures; ca. 3 μm thickness
	H_2_O/IPA (1:1 v/v)	Local aggregation of nanoribbon heads on nanograss surface; ca. 2 μm thickness
Silica source	TMOS	Dense and well-arrayed nanoribbon structures; ribbon with particulatelike and rough surface; ca. 2 μm thickness
	MS51	Dense and well-arrayed nanoribbon structures; ribbon with very smooth surface; ca. 3 μm thickness
Silica source concentration	50.0 vol %	Local aggregation of nanoribbon heads on the nanograss surface; ca. 1.5 μm thickness
	3.3 vol %	Highly dense and well-arrayed nanoribbon surface; ca. 3 μm thickness
	0.25 vol %	Relatively dense nanoribbons with slight surface aggregation; ca. 2 μm thickness
	0.1 vol %	No formation of nanograss structure, thin film with surface roughness; ca. 200 nm thickness
LPEI concentrations	3.0 wt %	Low-density array of ribbons with higher ratio of width to length; ca. 500 nm thickness
	5.0 wt %	Relatively high-density array of ribbons with lower ratio of width to length; ca. 1 μm thickness
Methanol addition for LPEI self-assembly	MeOH/H_2_O (3:7 v/v)	Ribbons with increased ratio of width to length (shorter ribbon); ca. 500 nm thickness.
	MeOH/H_2_O (9:1 v/v)	Nanowire-based network nanograss structure; ca. 300 nm thickness

### Control of the nanograss nanostructure by adjusting the media composition for silica deposition and using different silica sources

**Figure 2:**
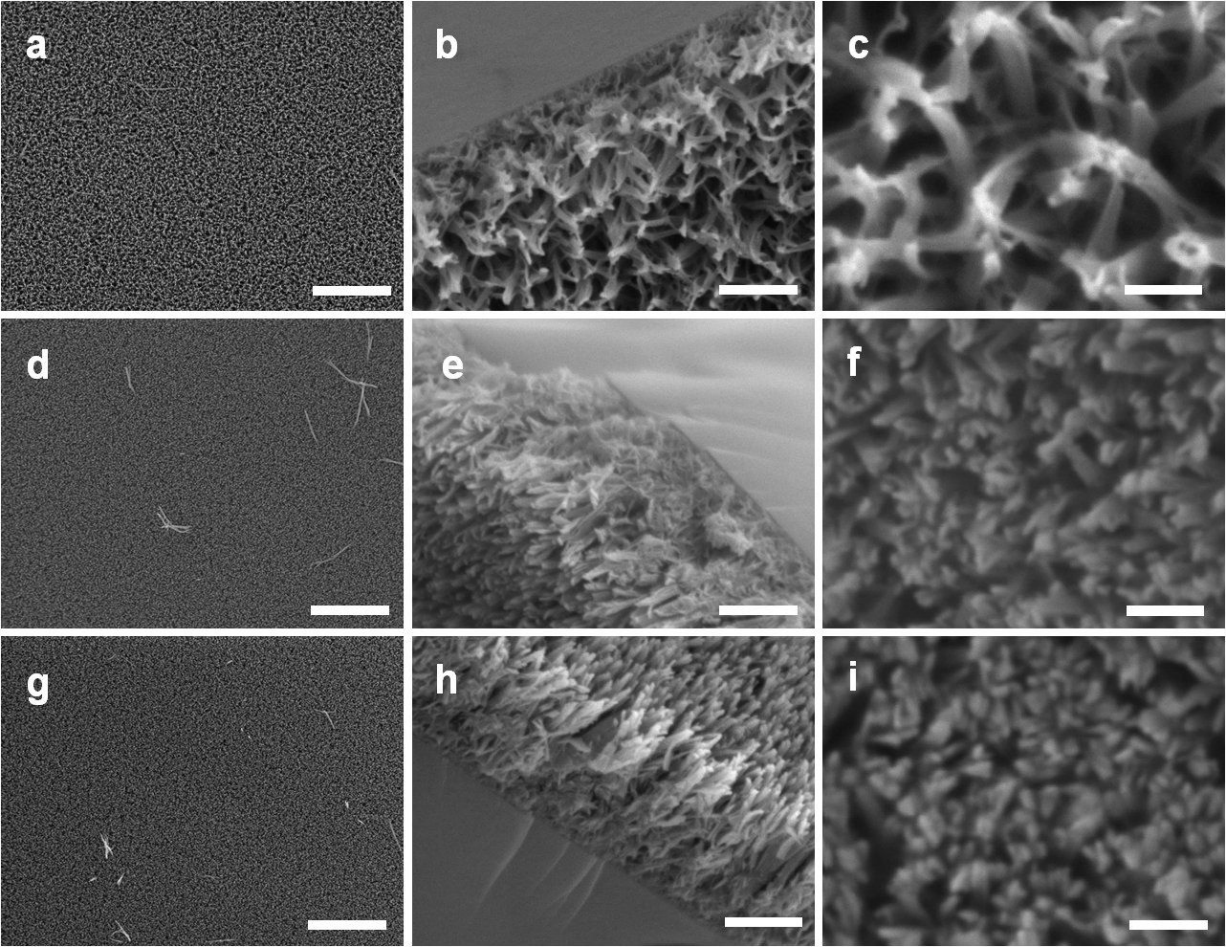
SEM images of LPEI@silica nanograss surface prepared from silica sources corresponding to the mixtures of 15 mL IPA, 15 mL water and 0.5 mL MS51 (a, b and c); 30 mL water and 0.5 mL MS51 (d, e and f); and 30 mL water and 0.5 mL TMOS (g, h and i). The LPEI matrix was formed from 5.0 wt % LPEI in water, and the silica deposition was performed for 40 min at room temperature. The scale bars are 10 μm (a, d and g); 1 μm (b, e and h); and 400 nm (c, f and i).

**Figure 3:**
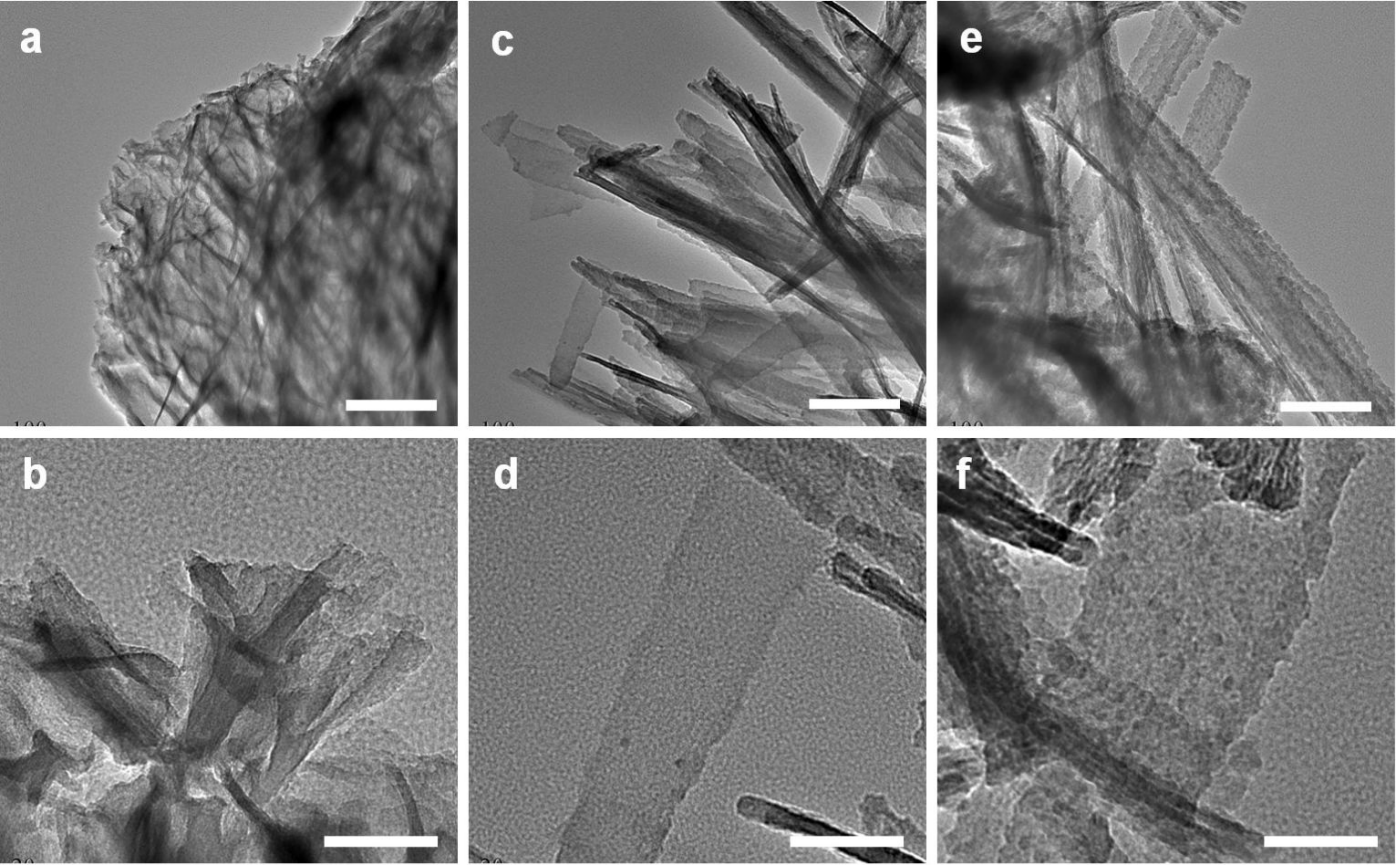
TEM images of LPEI@silica nanograss surface prepared from silica sources corresponding to the mixtures of 15 mL IPA, 15 mL water and 0.5 mL MS51 (a and b); 30 mL water and 0.5 mL MS51 (c and d); and 30 mL water and 0.5 mL TMOS (e and f). The LPEI matrix was formed from 5.0 wt % LPEI in water, and the silica deposition was performed for 40 min at room temperature. The scale bars are 200 nm (a, c and e) and 50 nm (b, d and f).

We found that the surface nanostructure of LPEI@silica nanograss can be controlled by adjusting the media composition for silica deposition. Figure 2a–c shows the SEM images of LPEI@silica nanograss film, which was synthesized by performing the silica deposition with a source of 0.5 mL MS51 in a mixed medium of 15 mL IPA and 15 mL water. The nanostructured LPEI matrix was formed from 5.0 wt % LPEI solution, and the silica deposition was conducted at room temperature for 40 min. We found that silicification in this mixture produced a highly uniform nanograss film (Figure 2a). The cross-section SEM image indicates a film thickness of about 2 μm (Figure 2b). TEM observation revealed that the hybrid nanograss has a fused film-like nanostructure composed of elemental nanoribbons (Figure 3a and b). We further found that the nanograss shows a very special surface morphology with a microscaled pyramidlike structure, which was formed by the fusion of the heads of several elemental nanoribbons (Figure 2c). A similar surface pattern has been observed for a carbon nanotube (CNT) film that was prepared by first depositing a CNT array on a silicon wafer and then performing PSS wrapping in water [35]. The formation of this CNT pyramidlike pattern was induced by the capillarity-driven self-assembly of preformed PSS-wrapped CNTs. In our case, the presence of IPA in the media was assumed to play an essential role in the formation of the pyramidlike structure since the use of neat water did not give this kind of surface structure. However, the detailed formation mechanism is still unclear.

Figure 2d–f shows the SEM images of a LPEI@silica nanograss film prepared from a silica source of 0.5 mL MS51 in a 30 mL water medium, with the other conditions identical to those used for silica deposition in the IPA–water mixture medium. We found that the silica deposition successfully occurred in this pure water medium, leading to the uniform LPEI@silica nanograss film (Figure 2d). In contrast to the nanograss formed in IPA–water mixture medium, the silica mineralization in the pure water medium generated a hybrid film consisting of much denser and well-arrayed nanoribbon structures (Figure 2f). There was no aggregation of nanoribbon heads on the film surface. TEM images indicate that this nanograss is composed of well-defined nanoribbons with a typical width of about 50 nm and a very smooth surface structure (Figure 3c and d). Cross-sectional observation showed a nanograss thickness of around 3 μm (Figure 2e), which is much thicker than that of the structures formed from the IPA–water mixture medium.

To assess the dependence of nanograss formation on the different silica sources, we performed a silica deposition on the nanostructured LPEI matrix by using TMOS as the source. The other conditions were the same as those used for nanograss formation from MS51 source in pure water (Figure 2d–f). As shown in Figure 2g–i, SEM observation demonstrated the successful formation of a high-quality nanograss surface, with a surface nanostructure similar to that prepared with MS51 as the silica source. However, the TEM study illustrates the obvious difference in the surface structure of the ribbon film. The ribbons from TMOS source show a particulatelike and rough surface (Figure 3e and 3f), whereas the use of MS51 as the source led to ribbons with a very smooth surface (Figure 3d). This reason for the difference in the surface structure was assumed to be the different rate of hydrolysis and polycondensation of TMOS and MS51. MS51 is silicic acid methyl ester as a tetramer on average. Thus, compared to TMOS, the silicification from MS51 under comparative conditions would be relatively slow and mild, which results in the formation of silica ribbons with a quite smooth surface nanostructure. Moreover, it should be noted that MS51 is safer and easier to handle than TMOS in industrial applications. While there have been many reports on biomimetic silica syntheses from various sources [13], studies comparing the nanostructures are very rare. We expect that our result on surface nanostructure control by means of different silica sources could be applied to other systems involving polyamine-mediated silica mineralization for the synthesis of silica with different structural features [36].

### Modulation of nanograss structure by changing the concentration of the silica source

**Figure 4:**
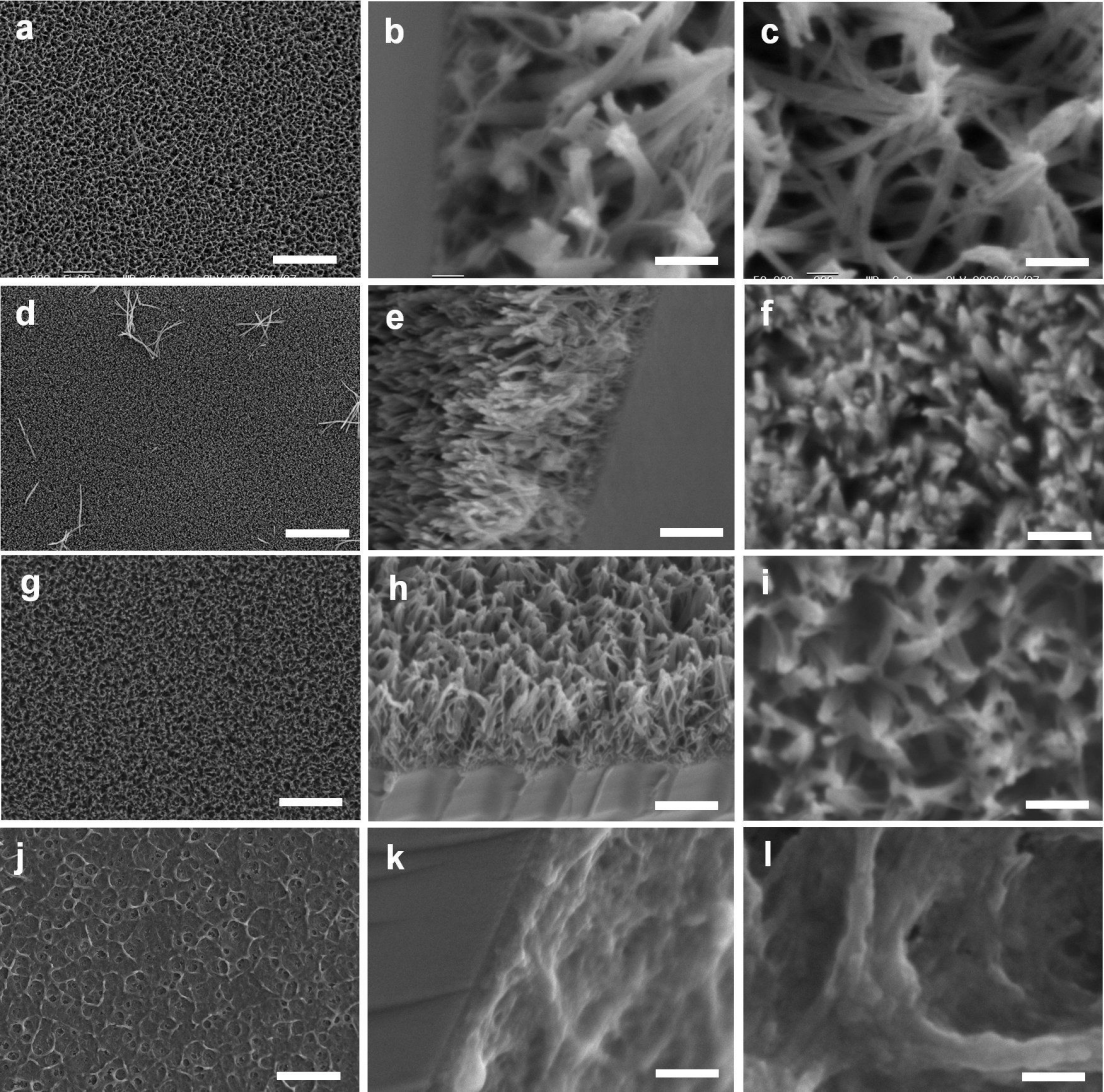
SEM images of the LPEI@silica nanograss surface obtained from silica sources with different concentrations: 50 vol % (3 mL TMOS and 3 mL water; a–c); 3.3 vol % (0.5 mL TMOS and 15 mL water; d–f); 0.25 vol % (0.25 mL TMOS in 100 mL water, g–i) and 0.1 vol % (0.1 mL TMOS in 100 mL water, j–l). The LPEI concentration is 5.0 wt % and the silica deposition was conducted at room temperature for 40 min. The scale bars are 10 μm (a, d, g and j); 1 μm (b, e, h, k and c); and 400 nm (f, i and l).

The surface nanostructure of the LPEI@silica nanograss was further found to be highly tunable by simply changing the concentration of the silica source. Figure 4 shows the SEM images of LPEI@silica nanosurfaces prepared from TMOS as the source in pure water medium with concentrations ranging from 50 to 0.1 vol %. The concentration of the aqueous LPEI solution used for the formation of the LPEI nanostructured matrix was 5.0 wt %, and the silica deposition reaction was conducted at room temperature for 40 min. The low-magnification SEM images show that silica mineralization from four representative TMOS concentrations all led to the excellent formation of high-quality and uniform LPEI@silica hybrid nanosurfaces (Figure 4a, d, g and j). However, the further, close SEM observation revealed an obvious difference in the surface nanostructures of LPEI@silica hybrid nanograss from different source concentrations. When an aqueous mixture composed of 50 vol % TMOS was used, we obtained a nanograss film with significantly local aggregation of the heads of the arrayed nanoribbons (Figure 4c). In comparison, silicification reaction with 3.3 vol % TMOS in water produced the highly dense and well-arrayed nanograss surface without head aggregation of the nanoribbons (Figure 4f). The nanoribbons of this surface have a significantly decreased size, compared to those from the 50 vol % source solution. When the TMOS concentration was further decreased to 0.25 vol %, a surface having relatively dense nanoribbons with slight surface aggregation was obtained (Figure 4i). No nanograss structure was observed when the TMOS concentration was lowered to 0.1 vol % (Figure 4l). Clearly, this low-concentration source cannot deposit enough silica on the LPEI matrix to maintain the structural stability of the LPEI@silica nanograss after drying of the sample in air. Such excellent tunability of the surface nanostructure of the LPEI@silica nanograss through changes of the source concentration was further verified by using MS51 as silica source in pure water (Supporting Information File 1, Figure S1).

### Dependence of the nanograss structure on LPEI concentration and methanol addition for LPEI self-assembly

**Figure 5:**
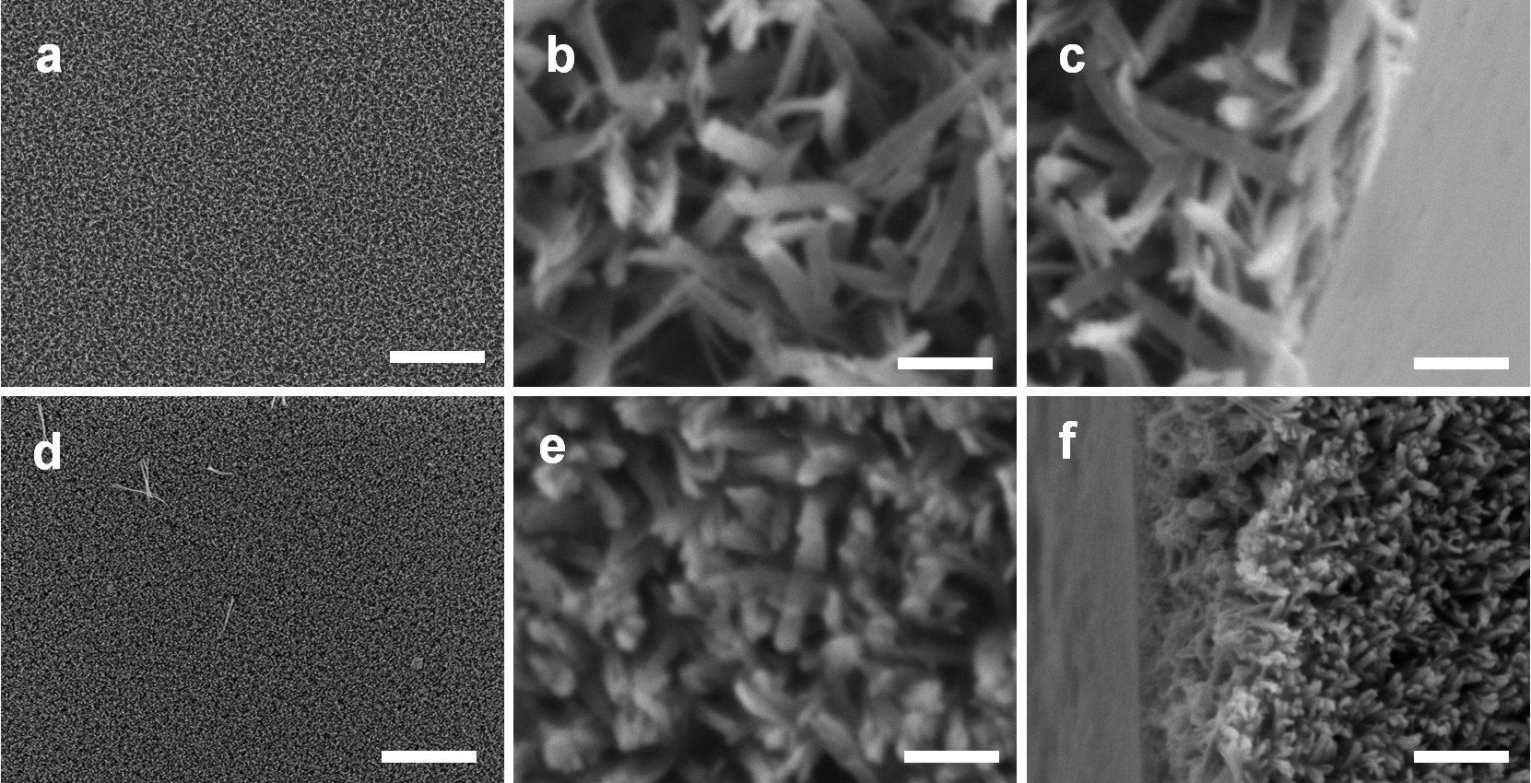
SEM images of LPEI@silica nanograss surface prepared from LPEI concentrations of 3.0 wt % (a, b and c) and 5.0 wt % (d, e and f). The silica-mineralization reactions were performed by using a source mixture of 15 mL water and 0.5 mL MS51 for 40 min at room temperature. The scale bars are 10 μm (a and d), 400 nm (b, c and e) and 500 nm (f).

LPEI concentration and methanol addition have been demonstrated to be efficient and simple methods to control the nanostructure and morphology of LPEI@silica hybrid materials in solution [29–30]. Here we found that the surface nanostructure of LPEI@silica hybrid nanograss can also be controlled by tuning the LPEI concentrations or adding methanol for LPEI crystalline self-assembly. Figure 5 shows the SEM images of LPEI@silica nanograss as prepared by mineralization of the nanostructured LPEI layer, which was formed from LPEI aqueous solutions with concentrations of 3.0 wt % (Figure 5a–c) and 5.0 wt % (Figure 5d–f). The silica-mineralization reaction was performed by using a source mixture of 15 mL water and 0.5 mL MS51 for 40 min at room temperature. The nanograsses from 3.0 and 5.0 wt % LPEI solutions both showed uniform and well-arrayed nanoribbon structures, without the local aggregation of ribbon heads. However, the two samples exhibited a remarkable difference in the density and width of the nanoribbons. The nanosurface resulting from the higher LPEI concentration (5.0 wt %, Figure 5d and 5e) showed a relatively high-density array of ribbons with a decreased width, compared to that from the low LPEI concentration (3.0 wt %, Figure 5a–c), as expected.

**Figure 6:**
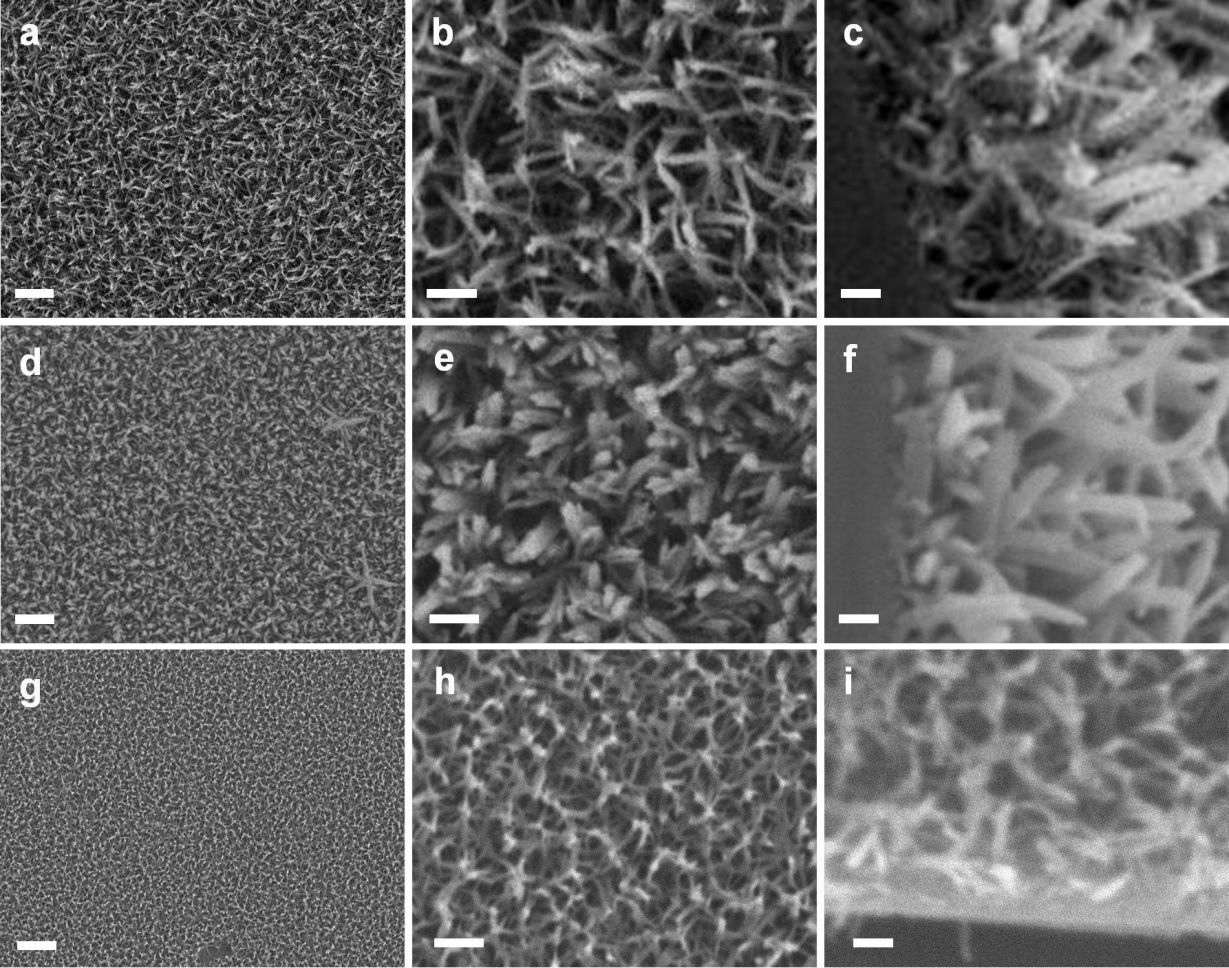
SEM images of LPEI@silica nanograss formed by using methanol as additive to control the LPEI self-assembly on the substrate surface. The self-assembled LPEI matrix was prepared from 4.0 wt % LPEI solutions in pure water (a–c) and in a mixture of methanol and water with volume ratio of 0.6 mL and 1.4 mL (d–f). The sample of g–i was prepared by a room-temperature process. The 3.0 wt % LPEI solution was prepared by dissolving LPEI into a methanol solution containing 10 vol % water. The tube with the film of LPEI in the methanol–water mixture was kept for 6 min at room temperature for methanol evaporation, leaving behind the self-assembled LPEI matrix. The silica depositions were performed in a mixture of 3 mL MS51 and 3 mL water for 40 min at room temperature for each of the three cases. The scale bars are 2 μm (a, d and g), 500 nm (b, e and h), 200 nm (c, f and i).

To address the possibility of using methanol addition to control the surface nanostructure of LPEI@silica nanograss, we compared the LPEI@silica nanograsses formed from 4.0 wt % LPEI in pure water (Figure 6a–c) and a methanol–water mixture of 3:7 (v/v) (Figure 6d–f). The silica deposition for both samples was the same and was performed in a mixture of 3 mL water and 3 mL MS51 at room temperature for 40 min. It was found that methanol addition led to the formation of LPEI@silica nanoribbons with an increased ratio of width to length, compared to those formed in pure water. This is consistent with the results of LPEI-mediated silica deposition in solution [30], where methanol addition was found to induce the formation of a ribbon structure. The addition of LPEI-dissolving methanol delays the crystallization process and this slow crystallization is beneficial for the formation of crystals with increased size.

Moreover, we also developed a methanol-mediated room-temperature process for LPEI thin-film self-assembly on the substrate for silica mineralization. A 3.0 wt % LPEI solution was first prepared by dissolving LPEI in a methanol solution containing 10 vol % water. LPEI does not crystallize in this methanol–water solution due to the relatively low water content (LPEI starts to crystallize when the water content is more than 50 vol %). This molecular LPEI solution was charged into tube, and then the excess solution was removed at room temperature, resulting in the methanol-based LPEI film formation on the inner wall of the tube. This tube was kept for 6 min at room temperature for natural evaporation of methanol, which induced the formation of the self-assembled LPEI matrix. Silica deposition was conducted in a mixture of 3 mL MS51 and 3 mL water for 40 min. As shown in Figure 6g–i, this methanol-mediated room-temperature process gave a high-quality nanograss surface composed of a nanowire-based network structure, with a film thickness of about 300 nm. This is different to those films formed by using hot LPEI solution in either pure water or water–methanol (3:7 v/v), both of which tended to form LPEI@silica with a ribbon nanostructure and with no local aggregation of nanoribbons observed (Figure 6a–f). This can be attributed to the differences in the formation mechanisms of the self-assembled LPEI matrices. Compared to the process of naturally cooling the hot LPEI solution in pure water or methanol-water (3:7 v/v), methanol evaporation would induce a relatively faster crystallization of LPEI in the thin film, leading to the formation of crystalline LPEI aggregates with decreased size and networklike structure between the nanounits. This room-temperature process for both LPEI matrix formation and silica deposition is important, because the heating process is undesirable for many biomimetic silica-derived technologies, such as the encapsulation of enzymes, cells or other temperature-sensitive biomolecules or systems [37–38].

### LPEI@silica nanograss with porphyrin function

**Figure 7:**
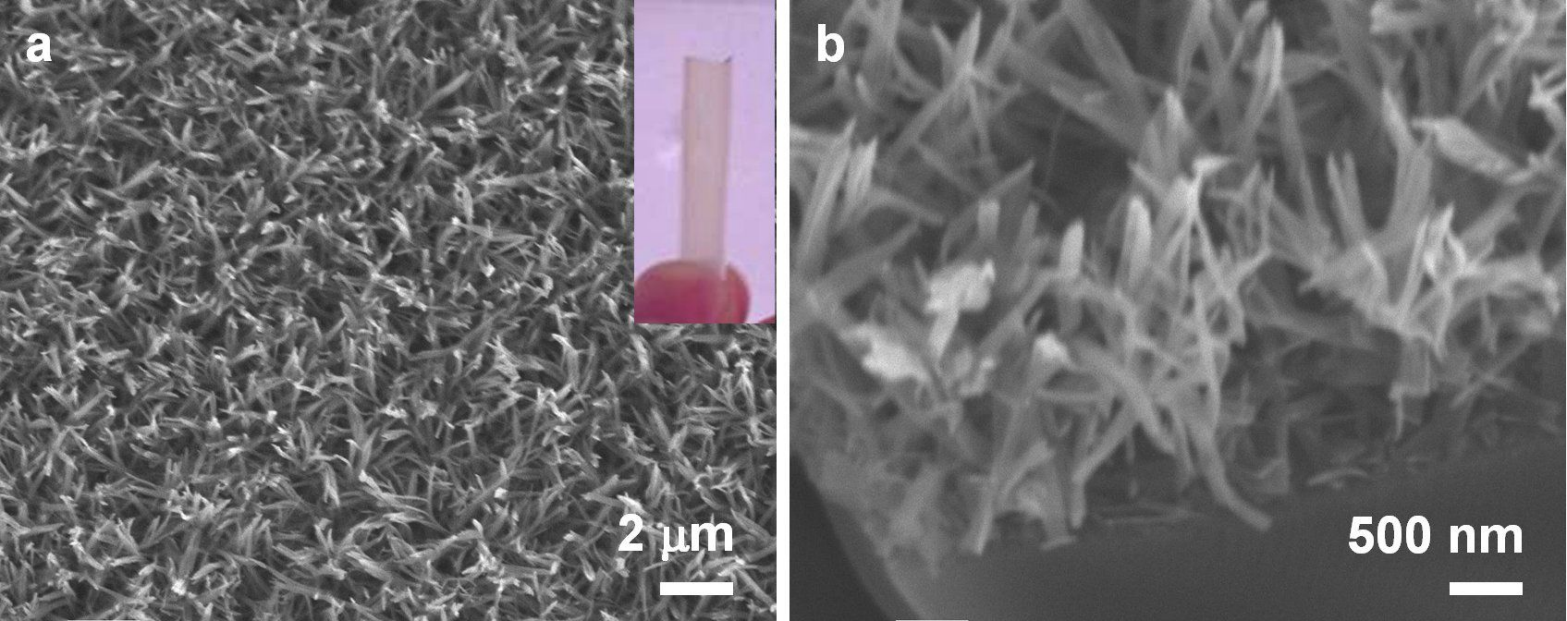
SEM images of LPEI@silica nanograss surface functionalized with porphyrin moieties. This functional nanograss was constructed by mineralization of silica on a self-assembled matrix prepared from a 3.0 wt % LPEI aqueous solution containing TSPP with the molar ratio of [EI]/[TSPP] = 300/1. A mixture of 3 mL IPA, 3 mL water and 0.2 mL MS51 was used as silica source and the silicification reaction was performed at room temperature for 40 min. The inset in (a) is a photograph of a tube with the functionalized nanograss, and the weak red colour indicates that the porphyrin has been successfully incorporated into the hybrid nanograss.

The ethyleneimine units of LPEI have a strong ability to associate with acidic molecules to form complexes by hydrogen-bond interaction. This simple acid–base complex chemistry has been exploited to control silica nanostructures in solution and further to achieve porphyrin functionalization of LPEI@silica powder materials with 5,10,15,20-tetrakis-(4-sulfonatophenyl)-21*H*,23*H*-porphyrin (TSPP) as an acidic functional molecule [30]. To examine the suitability of this idea for the incorporation of the TSPP into LPEI@silica nanograss, we first constructed the self-assembled LPEI matrix from a 3.0 wt % LPEI aqueous solution containing TSPP with a molar ratio of [EI]/[TSPP] = 300/1. This porphyrin-incorporating LPEI matrix appeared weak red in color due to the characteristic absorption by TSPP. After a silica deposition from a mixture of 3 mL water, 3 mL IPA and 0.2 mL MS51 for 40 min at room temperature, we obtained a functional porphyrin@LPEI@silica nanograss. This nanograss also showed a similar red color to that seen before silica deposition, indicating that the TSPP molecule was successfully incorporated into the LPEI@silica nanoribbon and was not released in the process of silica deposition. SEM studies demonstrated the formation of a high-quality nanograss surface with film thickness of about 2 μm and the arrayed ribbon nanostructure (as shown in Figure 7). In addition, we also attempted the use of LPEI solutions with increased molar ratios of ethyleneimine units to TSPP ([EI]/[TSPP] = 600/1 and 1200/1) for nanograss formation. Both were successful in the synthesis of the LPEI@silica nanograss with porphyrin functions. SEM images did not show any significant difference in the surface nanostructure with the change of molar ratio of [EI]/[TSPP] (Supporting Information File 1, Figure S2). Porphyrin-functionalized silica nanostructures in powder or colloidal form have been demonstrated to be important for bioimaging [39], sensors [40], cell photodynamic therapy [41] and biomimetic catalysis [42]. By growing porphyrin-based nanograss films directly on substrate surfaces, we expect that this new interface could be used in the fabrication of integrated optics or chips.

### LPEI@silica nanograss decorated with Au nanoparticles

**Figure 8:**
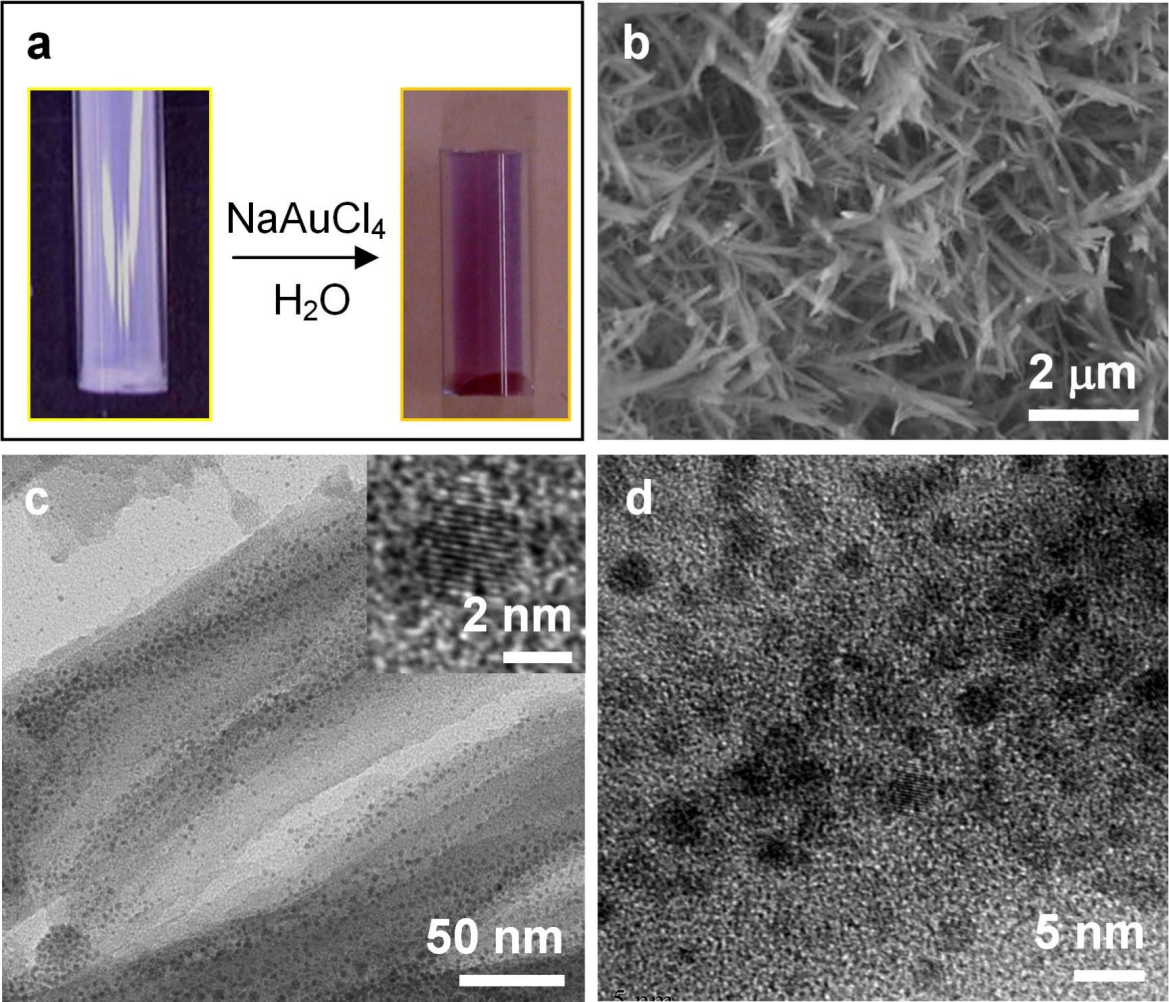
LPEI@silica nanograss surface decorated by Au nanoparticles. (a) Photographs of the nanograss on the inner surface of the tube before and after the formation of Au nanoparticles. SEM (b) and TEM (c and d) images of LPEI@silica@Au nanoparticles–nanograss surface synthesized by immersion of the LPEI@silica nanograss into an aqueous solution of NaAuCl_4_ (1.0 wt %) at 80 °C for 60 min. The inset of (c) is the high-resolution TEM image of a randomly selected Au nanoparticle, showing the well-resolved Au crystalline lattice.

Furthermore, we found that LPEI that has been hybridized into silica nanograss is also available for the functionalization of LPEI@silica nanograss. To confirm the chemical availability of LPEI in hybrid nanograss for in situ reduction of a metal ion, we immersed a tube with the inner wall coated with LPEI@silica nanograss (Supporting Information File 1, Figure S3) into an aqueous solution of NaAuCl_4_. After the reaction at 80 °C for 1 h, the nanograss film had changed to a red color (Figure 8a), indicating the successful generation of Au nanostructures on the LPEI@silica nanograss. SEM images show no damage or change to the surface of the LPEI@silica nanograss due to treatment in the aqueous solution of NaAuCl_4_, as seen before (Supporting Information File 1, Figure S3) and after Au nanoparticle formation (Figure 8b). TEM visualization indicated that Au nanoparticles were well-distributed over the whole nanoribbon structure, with a slight enrichment in the center part of the ribbon (Figure 8c), which probably reflects the presence of LPEI in the hybrid silica structure. HRTEM observation revealed that the Au nanoparticles had a typical diameter of 1–3 nm (Figure 8d) and a well-resolved crystalline lattice (Figure 8c inset). Therefore, similar to LPEI@silica powder materials [28,43], nanograsses on substrate surfaces also show the ability to serve as nanoreactors for the generation of metal nanoparticles, leading to a facile synthesis of functional silica-based nanosurfaces.

### Silica@titania composite nanograss and photoresponsive surface wettability

**Figure 9:**
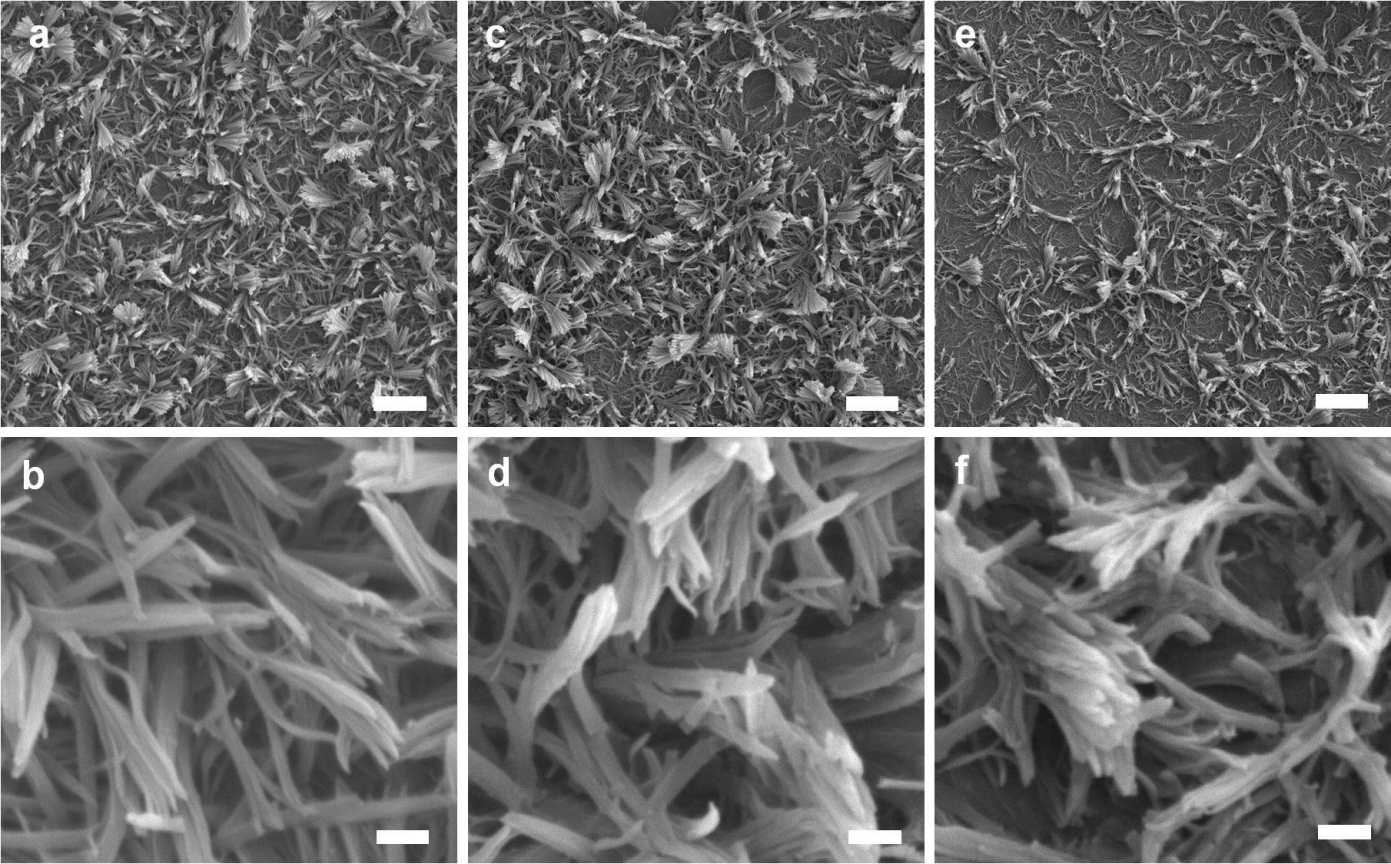
SEM images of nanograss surfaces of LPEI@silica (a and b), LPEI@silica @titania (c and d) and silica@titania (e and f). The scale bars are 5 μm (a, c and e) and 500 nm (b, d and f).

Finally, we attempted using LPEI in hybrid nanograss as a catalyst for titania deposition, with the aim to synthesize a silica@titania composite nanosurface after high-temperature calcination. We have previously shown that crystalline LPEI aggregates can be used as catalyst-active templates for the deposition of titania by using water-soluble titanium bislactates (TC315) as a source at room temperature, which led to the formation of LPEI@titania fibrous powder in solution [43] and a 50 nm nanowire-based surface on substrates [44]. Therefore, we expected that LPEI molecules in the LPEI@silica hybrid nanograss may be similarly active for the catalysis of titania deposition, producing the LPEI@silica@titania hybrid nanograss surface. To facilitate the evaluation of the sample surface composition and properties, the nanograss was prepared on a glass slide. Figure 9a and b shows the SEM images of the LPEI@silica nanograss formed on a glass slide, exhibiting good formation of the continuous and dense, fibrous nanosurface. It should be noted that the surface nanostructure of this nanograss on the glass slide is different to that formed in the inner wall of a soda lime glass tube, probably due to the differences in the substrate nature and/or shape. After titania deposition, no obvious change in the surface nanostructure was found, as judged by SEM observation (Figure 9c and d). This means that titania deposition selectively occurs on the template of the LPEI@silica nanograss, and that no nontemplated deposition takes place due to the absence of LPEI in the solution. Calcination of this LPEI@silica@titania at 500 °C resulted in the formation of a silica@titania composite nanograss surface. As shown in Figure 9e and f, the fibrous surface nanostructure is still clearly observable, although some local fusion appears. The titania deposition was further confirmed by Raman scattering. As shown in Supporting Information File 1, Figure S4, silica@titanis nanograss shows characteristic peaks at 197, 403, 505 and 637 cm^−1^, which are assumed to be associated with the anatase phase of titania [45].

**Figure 10:**
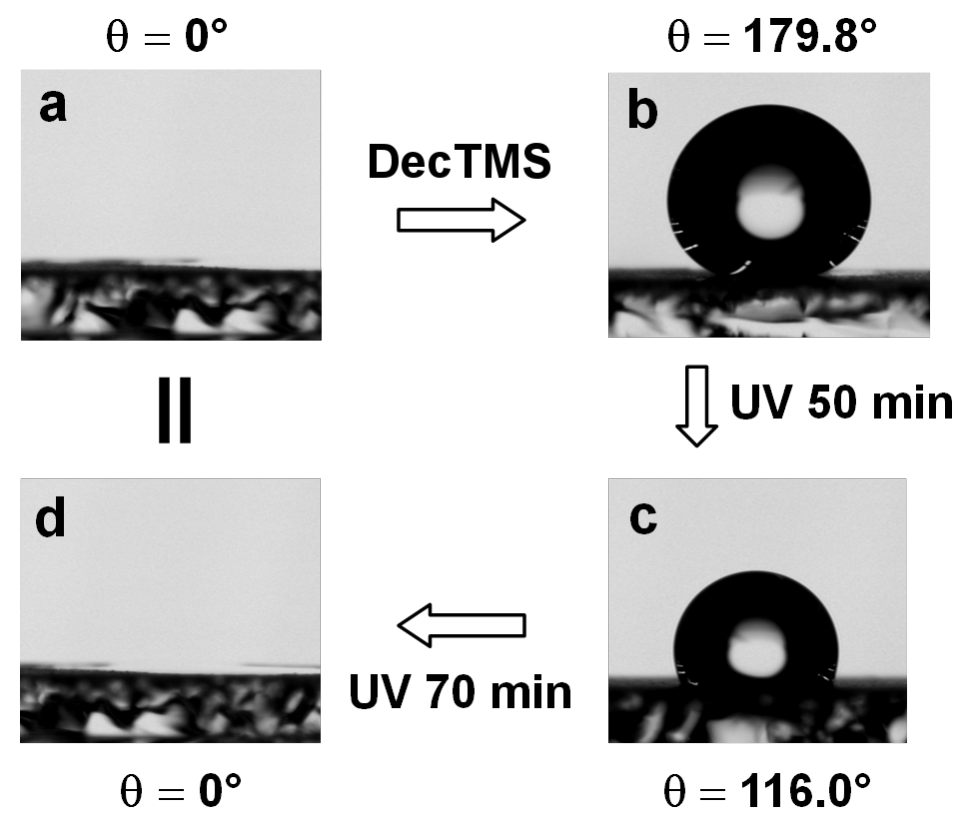
Water contact angles on the nanograss surface of (a) native superhydrophilic silica@titania, (b) superhydrophobic silica@titania after DecTMS modification, (c) hydrophobic silica@titania after 50 min UV irradiation and (d) superhydrophilic silica@titania after an additional 70 min irradiation (10 mW/cm^−2^).

We have previously shown that the titania coat composed of a 50 nm nanowire structure, which was prepared by a biomimetic deposition of titania directly on a self-assembled LPEI layer under ambient conditions, exhibits photoresponsive surface wettability through hydrophobic modification and light irradiation [44]. However, this surface failed to be superhydrophobic (i.e., water contact angle >150°) probably due to the relatively low surface roughness. In this work, by depositing the titania on the preformed LPEI@silica nanograss surface, we were able to construct a smart silica@titania composite nanosurface that could switch its wetting behavior from superhydrophobic to superhydrophilic state under UV irradiation (black light, 10 mW/cm^−2^). As shown in Figure 10, the native silica@titania composite nanograss surface was superhydrophilic (with θ = 0°) due to the high roughness and surface energy. After treatment with decyltrimethoxysilane (DecTMS) [46], the surface changed to become superhydrophobic with a water contact angle of about 179.8°. The water contact angles decreased to 116° and finally to 0° upon irradiating the superhydrophobic nanograss surface with black light for 50 min and an additional 70 min, respectively. SEM observation indicated that there was no change of surface nanostructure before and after UV treatment (Figure 9e and f). Therefore, the decrease of the water contact angle could be attributed to the degradation of the low-surface-energy alkyl group due to the photocatalytic activity of anatase titania [47]. This was further confirmed by a simple control experiment. A superhydrophobic silica nanograss surface was produced by a similar DecTMS treatment. After irradiating this surface under identical conditions to those used for the silica@titania composite nanosurface, we did not observe any decrease of the contact angle, due to the absence of the titania component in this control nanosurface (Supporting Information File 1, Figure S5). Thus, we believe that the photocatalysis-active anatase titania component in the silica@titania composite nanograss surface plays a vital role in the induction of the change of surface wettability, from a superhydrophobic to a superhydrophilic state.

## Conclusion

In summary, we have successfully demonstrated that the nanostructure and surface morphology of LPEI@silica nanograss can be well controlled by adjustment of a series of simple parameters for the silica-mineralization reaction and LPEI self-assembly on the substrate surface. This achievement did not require any complex macromolecule design on the surface or tedious procedures, unlike in the case of a previously reported biomimetic synthesis of a polymer@silica hybrid thin film [21–23]. Moreover, both free LPEI and the LPEI occluded in a hybrid silica nanostructure were successfully explored in order to introduce, separately, a porphyrin moiety, Au nanoparticles, and a titania component into the LPEI@silica hybrid nanograss, leading to the facile creation of a functional nanosurface. This novel functionalization concept could be generally applied to other polyamine@silica hybrid materials. The silica@titania composite nanograss has been demonstrated to be a novel external stimuli-responsive smart surface [47], with the superhydrophobic surface being switched into a superhydrophilic state by means of UV irradiation.

## Experimental

### Materials

LPEI with an average polymerization degree of around 505 was synthesized by the hydrolysis of the corresponding precursor poly(oxazoline)s (linear poly(ethyloxazoline): *M*_w_ = 50,000, *M*_w_/*M*_n_ = 1.9) in an aqueous solution of 5 M HCl at 100 °C for 12 h, according to our previous method [27]. MS51 (silicic acid methyl ester tetramer) and TC315 (aqueous solution containing ca. 40 wt % titaniumbislactate) were purchased from Colcoat Co. Japan and Matsumoto Chemical Co. Japan, respectively. Tetramethyl orthosilicate (TMOS), NaAuCl_4_ and poly(4-styrenesulfonic acid) (PSS, *M*_w_ = 75,000, 18 wt % in water) were purchased from Aldrich. Decyltrimethoxysilane (DecTMS) was provided by Shinetsu Co. Japan. All chemicals were used as received. Deionized water was used in all experiments.

### LPEI@silica hybrid nanograss on the inner surface of soda lime glass tubes

Pasteur pipettes of soda-lime glass (IK-PAS-5P, Asahi glass Co. Japan) were used as representative substrates for nanograss growth. The 3.0–5.0 wt % LPEI hot solution was prepared by dissolving LPEI powder into water at 80 °C. The assembly of a LPEI layer on the inner surface of the tubes was simply achieved by first charging the hot aqueous solution of LPEI into the tube, and then by removal of the excess hot solution from the tube immediately, to leave a tube with the inner wall coated with an aqueous-solution layer of molecular LPEI (mLPEI@tube). This LPEI hot-solution-treated tube was left for 5–10 min at room temperature for complete formation of a crystalline, self-assembled LPEI layer before silica deposition (cLPEI@tube). The silicification was performed by immersing the cLPEI@tube into the silica source solution, and the silica deposition reaction was allowed to proceed at room temperature for typically 40 min. The final LPEI@silica nanograss on the inner wall of the tube was obtained by washing with ethanol and natural drying.

### Functionalization of LPEI@silica nanograss

Porphyrin-functionalized LPEI@silica nanograss was constructed by mineralizing silica on a self-assembled LPEI matrix that already incorporated the porphyrin moiety. The solution used for the formation of the matrix was prepared by adding TSPP into a 3.0 wt % LPEI aqueous solution at a molar ratio of [EI]/[TSPP] of 300/1. A mixture of 3 mL IPA, 3 mL water and 0.2 mL MS51 was used as silica source and the silicification reaction was performed at room temperature for 40 min. UV–vis and fluorescence spectra indicated the isolated presence of porphyrin moiety in LPEI@silica hybrid by using a powder sample, which was synthesized by performing silica deposition in solution with the same porphyrin-LPEI aggregates [30].

The formation of Au nanoparticles in the LPEI@silica nanograss was simply achieved by immersing the LPEI@silica nanograss into the aqueous solution of NaAuCl_4_ (1.0 wt %) at 80 °C for 60 min. LPEI@silica nanograss decorated with Au nanopartilces was obtained by washing with water and natural drying, and is red in color due to the plasma absorption by the Au nanoparticles. UV–vis spectrum also showed the absorption at around 580 nm, indicating the formation of Au nanoparticles (Supporting Information File 1, Figure S6).

The titania deposition was conducted by immersing the LPEI@silica nanograss into a diluted aqueous titania source solution consisting of 0.2 mL TC315 and 50 mL water. The silica@titania composite nanograss was obtained after 3 h deposition at room temperature, followed by water washing and naturally drying. The silica@titania composite nanograss was obtained by calcining the LPEI@silica@titania sample in air to 500 °C at a heating rate of 2 °C per min and maintaining this temperature for 3 h in order to completely remove the organic polymer. It should be noted that, to facilitate the surface wettability study, the LPEI@silica hybrid nanograss on a glass slide was used, which was prepared by a two-step dipping process, as described previously [33]. The LPEI layer was formed by briefly dipping the substrates into 3 wt % hot aqueous solution (80 °C) of LPEI. The hot substrates covered with aqueous LPEI were kept for 20–30 s at 20 °C in air for the formation of nanostructured layer of crystalline LPEI.

The superhydrophobic property of LPEI@silica, silica (after calcination at 500 °C) and silica@titania nanograss was simply achieved by introducing lower-free-energy residues of silane coupling agents into the films. The silane introduction was performed by following the procedure reported by Wang et al., with some modifications [46]. Typically, the nanograss-covered slide (2.5 × 3.5 cm) was immersed into a mixture prepared by adding a 3 mL decyltrimethoxysilane chloroform solution (20% w/v) into 30 mL of an ethanol solution containing 0.6 mL of NH_3_-H_2_O (28%), under stirring. The reaction was carried out at room temperature for 1–24 h. The superhydrophobic nanograss film was obtained by rinsing with ethanol and drying under N_2_ flow.

### Characterizations

The surface morphology and nanostructured grass were observed by means of scanning electron microscopy (Kyence, VE9800, Japan, working at 8 kV) and field-emission scanning electron microscopy (JEOL JSM-7500F, working at 15 kV). The samples were sputter-coated with a thin overlayer of Pt prior to observation. Transmission electron microscopy (TEM) studies were conducted on a TEM instrument (JEOL JEM-2200FS) operating at 200 kV. The water contact angle (WCA) was measured by using an optical contact angle meter (OCA20, DataPhysics, Germany) with liquid drop of 5 μL.

## Supporting Information

File 1 Additional figures
