# Supplementary material for: Approaches to nanostructure control and functionalizations of polymer@silica hybrid nanograss generated by biomimetic silica mineralization on a self-assembled polyamine layer
Source: Beilstein J Nanotechnol. 2011 Nov 23;2:760–73. doi: 10.3762/bjnano.2.84 (PMC3257501; doi:10.3762/bjnano.2.84)
Supplement: File 1 — Additional figures [file Beilstein_J_Nanotechnol-02-760-s001.pdf]

Supporting Information

for

Approaches to nanostructure control and functionalizations of polymer@silica hybrid nanograss surface generated by biomimetic silica mineralization on a self-assembled polyamine layer

Jian-Jun Yuan[1] and Ren-Hua Jin*[1,2]

Address: [1]Synthetic Chemistry Lab., Kawamura Institute of Chemical Research, 631 Sakado, Sakura, 285-0078 Japan and [2]CREST-JST, 631 Sakado, Sakura, Chiba 285-0078, Japan

* Corresponding author

Correspondence and requests for materials should be addressed to R.H.J

(Tel:+81-43-498-4104, Fax:+81-43-498-2202, jin@kicr.or.jp)

Additional figures

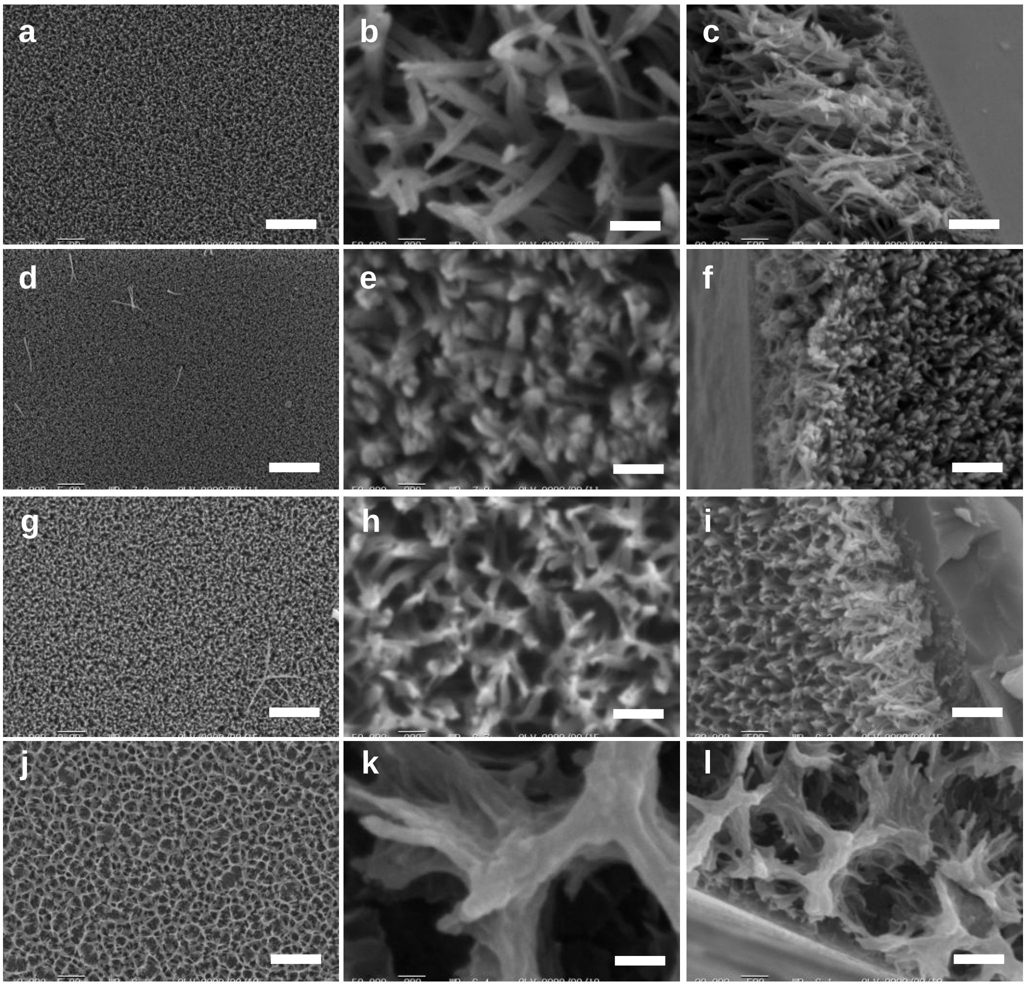

Figure S1: SEM images of LPEI@silicas nanograss surface obtained by using the silica sources of MS51 with different concentrations: 50 vol % (3 mL MS51 and 3 mL water; a–c); 3.3 vol % (0.5 mL MS51and 15 mL water; d–f); 0.25 vol % (0.25 mL MS51in 100 mL water, g–i) and 0.1 vol % (0.1 mL MS51 in 100 mL water, j–l). The LPEI concentration is 5.0 wt % and the silica deposition was conducted at room temperature for 40 min. The bars are 10 μm for a, d, g and j; 400 nm for b, e, h and k, 1 μm for c, f, i and l.

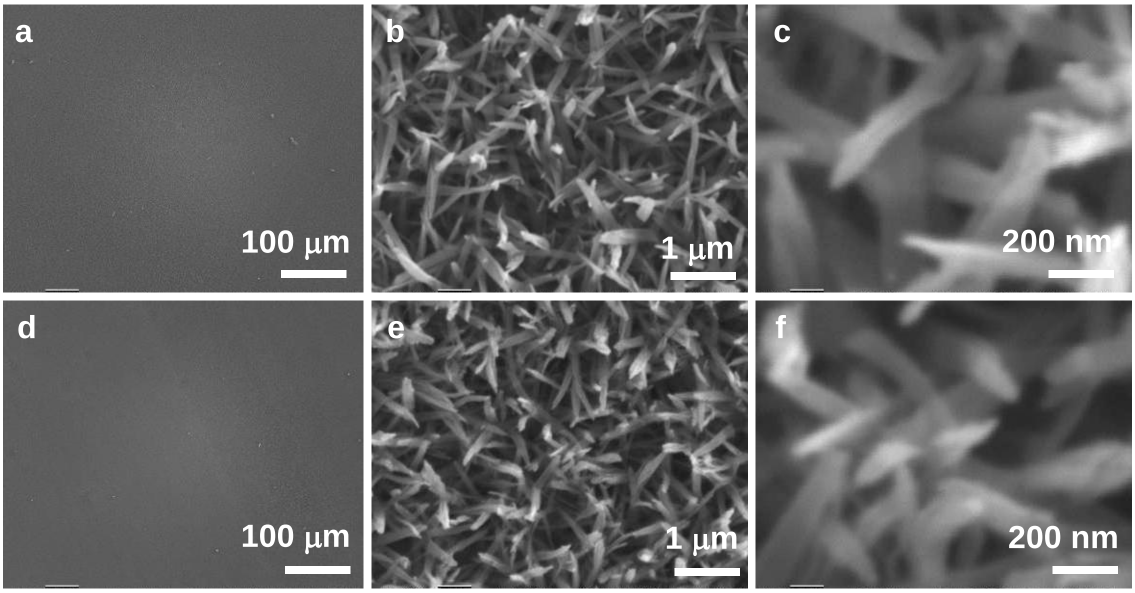

Figure S2: SEM images of LPEI@silica nanograss surface functionalized with porphyrin moiety with molar ratio of [EI]/[TSPP] = 600/1 (a–c) and 1200/1 (d–f). The other synthesis conditions are the same as those used for the sample shown in Figure 7.

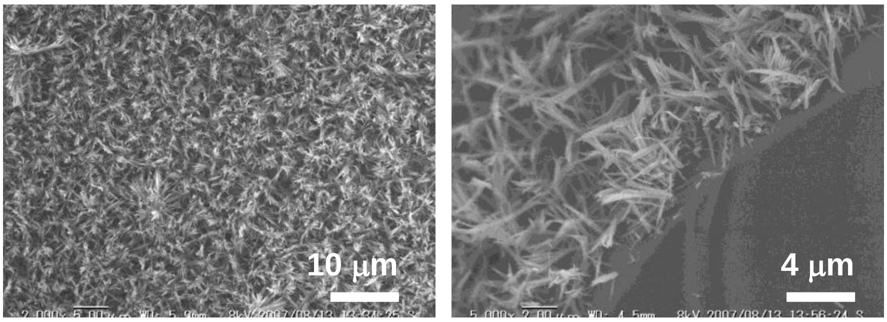

Figure S3: SEM images of LPEI@silica nanograss surface used for the generation of Au nanoparticles.

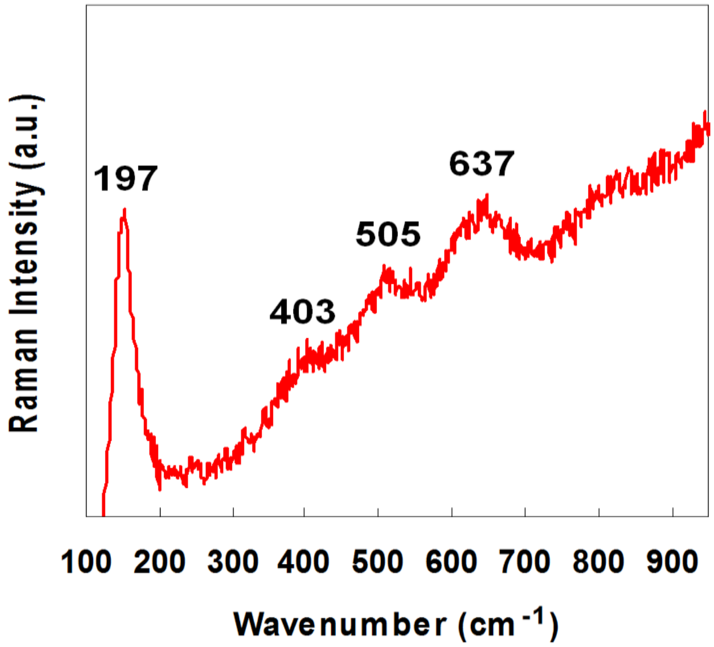

Figure S4: Raman spectrum of silica@titania composite nanograss surface.

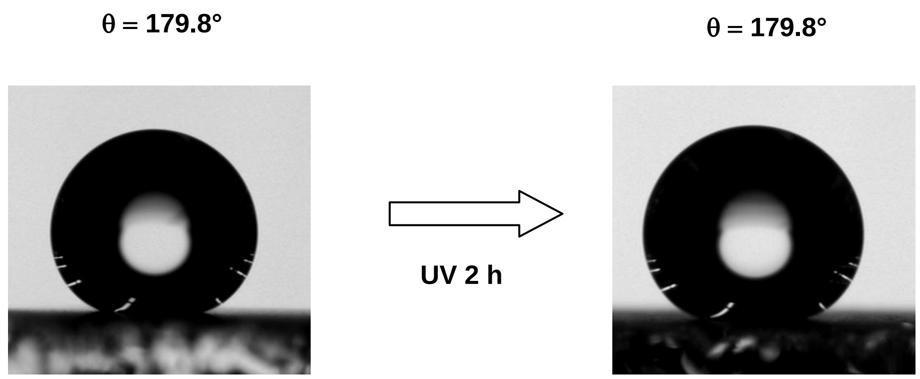

Figure S5: Water contact angles of silica nanograss surface (as shown in Figure 9a and b) before and after 2 h UV irradiation.

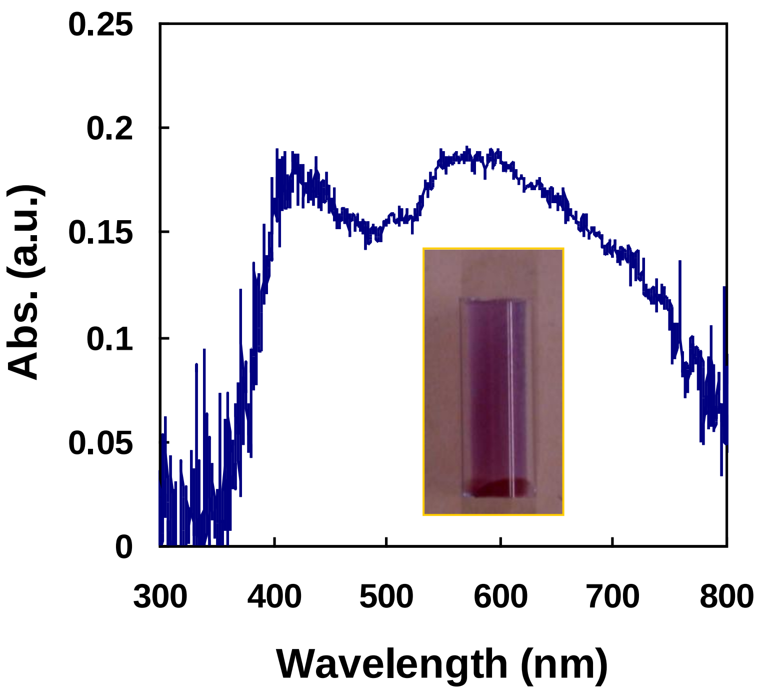

Figure S6: UV–vis spectrum of LPEI@silica nanograss functionalized with nanoparticles.
